# Validation of a risk prediction score for proximal neoplasia in colorectal cancer screening: a prospective colonoscopy study

**DOI:** 10.1038/srep20396

**Published:** 2016-02-08

**Authors:** Martin C.S. Wong, Jessica Y.L. Ching, Victor C.W. Chan, Raymond S.Y. Tang, Arthur K.C. Luk, Thomas Y.T. Lam, Sunny S.H. Wong, Siew C. Ng, Simon S.M. Ng, Justin C.Y. Wu, Francis K.L. Chan, Joseph J.Y. Sung

**Affiliations:** 1 Institute of Digestive Disease, Chinese University of Hong Kong, Shatin, Hong Kong, HKSAR, China; 2 School of Public Health and Primary Care, Chinese University of Hong Kong, Shatin, Hong Kong, HKSAR, China

## Abstract

This study developed a clinical scoring system to predict the risks of PN among screening participants for colorectal cancer. We recruited 5,789 Chinese asymptomatic screening participants who received colonoscopy in Hong Kong (2008–2014). From random sampling of 2,000 participants, the independent risk factors were evaluated for PN using binary regression analysis. The odds ratios for significant risk factors were used to develop a scoring system, with scores stratified into ‘average risk’ (AR):0–2 and ‘high risk’ (HR):3–5. The other 3,789 subjects formed an independent validation cohort. Each participant received a score calculated based on their risk factors. The performance of the scoring system was evaluated. The proportion of PN in the derivation and validation cohorts was 12.6% and 12.9%, respectively. Based on age, gender, family history, body mass index and self-reported ischaemic heart disease, 85.0% and 15.0% in the validation cohort were classified as AR and HR, respectively. Their prevalence of PN was 12.0% and 18.1%, respectively. Participants in the HR group had 1.51-fold (95% CI = 1.24–1.84, p < 0.001) higher risk of PN than the AR group. The overall c-statistics of the prediction model was 0.71(0.02). The scoring system is useful in predicting the risk of PN to prioritize patients for colonoscopy.

Colorectal Cancer (CRC) is the second commonest cancer worldwide, accounting for 10% of all malignancies and 8% of cancer mortality^1^. Its incidence and mortality in Asia, with more than 4 billion people, continues to rise in an alarming rate without signs of decline^2^,^3^. Flexible sigmoidoscopy (FS) and colonoscopy are two common screening tests for CRC, and were found effective to reduce CRC mortality by 40% and 68%, respectively^4^,^5^,^6^. A joint guideline from the American Cancer Society, the US Multi-Society Task Force on Colorectal Cancer, and the American College of Radiology recommended that CRC prevention by polyp detection and removal should be the primary goal of screening^7^. The guideline also highlighted the importance of making an informed choice, allowing patients to appreciate the various screening options, as well as their pros and cons^7^.

FS is now more popularly perceived, especially in European countries – following the publication of findings from landmark randomized trials^8^,^9^,^10^,^11^. A recent meta-analysis reported that both FS and colonoscopy prevent most deaths from distal CRC, but reduction in deaths from proximal CRC was only observed in those who received colonoscopy^12^. Nevertheless, colonoscopy is relatively invasive, labor-intensive, and expensive. Its demand is increasing, and endoscopic capacity constraints induce a prohibitive challenge to population-based CRC screening^13^,^14^. It also requires a high level of expertise-which implies that it might not be appropriate as a first-line test in regions with deprived resources. On the contrary, FS is an office-based procedure requiring minimal bowel preparation and no sedation. It represents an attractive option as it is safe, and can be conducted by trained personnel without a medical license^15^. It is presently the screening test of choice in organized programmes in many countries.

The choice between FS and colonoscopy should be based on individual risks of proximal neoplasia (PN). Thus far, there have been few validated tools which could be used clinically to predict PN^16^,^17^,^18^,^19^, and none exists for screening participants in Asian countries. We have previously evaluated the factors associated with PN and distal neoplasia (DN), as well as assessed the use of the US, UK, Italy, Norwegian and a Hong Kong criterion to predict PN among asymptomatic screening participants^20^. All of the existing scoring systems were reported to have limited predictive and discriminative ability. There is an urgent need to devise a validated risk prediction system for the Asian population.

The primary objective of this study was to develop and validate a clinical risk stratification score predicting the risk of PN among asymptomatic subjects. We aim to construct a simple tool for physicians so that information on risks could be easily computed to inform choice of screening options. Our secondary objective is to validate a similar system, with synchronous presence of PN and DN as the outcome of interest.

## Methods

The study setting has been described elsewhere^21^,^22^,^23^,^24^,^25^,^26^. In short, a community CRC screening centre was established in Hong Kong in 2008. Through several territory-wide media invitations, it invited free CRC screening for all eligible Hong Kong residents. The study was approved by the Clinical Research Ethics Committee of the Chinese University of Hong Kong (protocol CRE-2008.404). The methods were carried out in accordance with the approved guidelines. All participants provided informed consent for the study.

### Study Participants

The screening participants voluntarily enrolled for the programme via online application, telephone, e-mail, fax or walk-in. The inclusion criteria consist of: (i) Age between 50 to 70 years; (ii) The absence of current or previous symptoms suggestive of CRC, such as per rectal bleeding, tarry stool, anorexia or a change in bowel habit in the past 4 weeks, or a weight loss of greater than 5 kg in the past 6 months, and (iii) Not having received any CRC screening tests in the past 5 years. Exclusion criteria included: (i). A personal history of CRC, colonic adenoma, diverticular disease, inflammatory bowel disease, prosthetic heart valve or vascular graft surgery, and (ii). Medical conditions which were contraindications for colonoscopy, like cardiopulmonary insufficiency and the use of double antiplatelet agents. The eligibility of each participant was checked by trained staff in the centre.

Each eligible participant completed a self-administered questionnaire, which consists of information on their age, gender, family history of CRC, smoking status, drinking habits, past medical history, and chronic use of medications. Meanwhile, the completeness of questionnaires was checked by trained personnel. For relatively illiterate participants, trained volunteers assisted with survey completion.

Each participant was offered a choice between receiving FIT yearly for up to five years, or one direct colonoscopy. The present study included all screening participants who have given informed consent and received colonoscopy in this programme.

### Colonoscopy procedure

The detailed procedure of colonoscopy was explained to each study participant before the scheduled colonoscopy appointment. Polyethylene Glycol (Klean-Prep^R^, Helsinn Birex Pharmaceuticals Ltd, Ireland) in split-dosing was used as a standardized bowel preparation regimen, which were given to all participants before they left the centre. Colonoscopy was performed by experienced colonoscopists in endoscopy centres affiliated with two major hospitals. Prior to the procedure, all subjects received a sedation regimen consisting of Midazolam 2.5 mg (Groupe Panpharma, France). Meperidine 25 mg (Martindale Pharmaceuticals, United Kingdom) was administered intravenously. Further doses of midazolam and meperidine were supplemented subject to the needs of the participants. Air insufflation was used for all procedures. The endoscopists aimed for a withdrawal time of ≥6 minutes, according to the current quality indicators for colonoscopy. As deemed appropriate by the endoscopists, lesions were removed and biopsied. The specimens were sent to a certified, accredited laboratory for gross and histopathological examination.

### The derivation and validation cohort

A total of 5,789 screening participants completed colonoscopy in the study period 2008–2012. Among them, simple random sampling was used to select 2,000 subjects to act as the derivation cohort – a methodology based on our previous validation study^21^. Each study participant was regarded as one unit of randomization, and had an equal probability of being selected. “Proximal” refers to a location in the colon which is proximal to the splenic flexure; whilst “distal” refers to the rectum, sigmoid and descending colon. The proportion of PN in the derivation cohort was 12.6%, and we assumed 25% as the point prevalence of individual risk factors, as in the Asia Pacific Colorectal Screening (APCS) study by Yeoh and colleagues^27^. Based on these assumptions, a minimum of 3,100 subjects were needed to attain a power of >80% so that a risk factor with an odds ratio of two could be detected at a significance level of p < 0.05. Therefore, the other 3,789 subjects formed our validation cohort.

### Development of the risk scores

In the derivation cohort, the association between the colonoscopic finding of PN and each risk factor was examined by the Pearson Chi-square tests. The risk factors examined included age, sex, family history of CRC (in first-degree relatives before the age of 60 years)^7^, smoking, drinking (current drinkers of alcohol for more than two times per week vs. those drinking less or non-drinkers), Body Mass Index (BMI), self-reported medical conditions, and use of non-steroidal anti-inflammatory agents (NSAIDs) or aspirin. All variables with initial p < 0.05 in univariate analysis were included in a binary logistic regression model with PN as the outcome. As was adopted by Yeoh and colleagues^28^, a weighting was assigned to each independent variable in the risk score, applying the corresponding adjusted odds ratio (AOR) halved and rounded to the nearest integer. This statistical technique aims to keep the total score below ten, and make the scoring system simple. The risk score for each subject is the sum of all the risk factors. To evaluate the predictive ability of the scoring system, a receiver operating characteristic (ROC) curve was constructed and the area under the curve (AUC) was delineated. A concordance (c)-statistics was used to reflect the discriminative ability of the prediction tool.

### Statistical analysis

The Statistical Package for Social Science (SPSS) version 16.0 (Chicago, Illinois) was used for data analysis. The proportion of PN was evaluated according to each score in the derivation cohort. The score with a magnitude closest to and below the overall proportion of PN was allocated to the category “average risk”, whilst scores above were assigned as “high risk”. An additional binary logistic regression model was constructed by entering all the significant variables identified by the derivation cohort analysis, and the AORs were evaluated in the validation cohort. The Hosmer-Lemeshow goodness-of-fit statistic was adopted to assess the reliability of the final model, where p > 0.05 indicates a good match of predicted risk over observed risk. C-statistics and the area under the ROC curve were used to evaluate the ability of the scoring system to predict the risk of having PN. The Cochran-Armitage test of trend was used to compare the prevalence of PN according to scores. The above analyses were repeated with synchronous presence of PN and distal neoplasia (DN) as the outcome of prediction. P values (two-sided) <0.05 were considered statistically significant.

## Results

### Subject characteristics

In the derivation cohort, the average age of the participants was 57.8 years (SD 4.9) with 47.2% being male subjects (Table 1). A total of 645 (32.2%) cases of colorectal neoplasia were detected, including 8 (0.4%) and 122 (6.1%) being CRC and advanced neoplasia, respectively. The proportion of subjects having PN, DN, and synchronous presence of PN/DN was 12.6%, 14.9% and 4.7%, respectively. The characteristics of the validation cohort were similar to the derivation set, except BMI (p = 0.043). The prevalence of colorectal neoplasia according to the risk factors is shown in Table 2. From univariate analysis, age (p = 0.001), gender (p = 0.001), family history (p = 0.035), BMI (p = 0.013), and self-reported ischemic heart disease (p = 0.019) were associated with PN. For synchronous PN/DN, the factors identified included age (p < 0.001), gender (p < 0.001), smoking (p = 0.008), alcohol drinking (p = 0.017), and BMI (p = 0.017) (Table 2).

### Independent predictors of proximal neoplasia in the derivation cohort

From binary logistic regression analysis, advancing age for each 5 year period from 50 years onwards (AOR 1.5 to 1.8), male gender (AOR 1.5, 95% C.I. 1.2–2.0, p = 0.002), a positive family history in a first degree relative (AOR 1.5, 95% C.I. 1.1–2.2, p = 0.016), BMI ≥ 25 (AOR 1.3, 95% C.I. 1.0–1.8, p = 0.047), and ischaemic heart disease (IHD) [AOR 2.2, 95% C.I. 1.0–4.8, p = 0.048] were significantly associated with PN (Table 3). Age (AOR 1.3 to 3.4), gender (AOR = 3.1, 95% C.I. 1.9–5.0, p < 0.001) and BMI (AOR = 1.6, 95% C.I. 1.0–2.5, p = 0.042) were significant independent predictors of synchronous PN/DN (Table 4).

### Development of the risk scoring systems

According to the AORs from the derivation cohort, the following predictors of PN were used to assign scores to each subject (Table 5): age 50–55 years (0), 56–70 (1), male gender (1), female gender (0), family history of CRC in a first-degree relative present (1) or absent (0), BMI < 25 kg/m^2^ (0), BMI ≥ 25 kg/m^2^ (1), self-reported IHD (1), no IHD (0). The assignment of scores for synchronous PN/DN was (Table 6): age 50–55 years (0), 56–65 (1), 66–70 (2), male gender (2), female gender (0), BMI < 25 kg/m^2^ (0), BMI ≥ 25 kg/m^2^ (1). For both systems, the range of scores was 0–5, and a participant’s score was based on the summation of all the points assigned to each risk factor. The number and proportions of subjects having various scores were shown in Tables 7. Since a score of 2 had a proportion of PN closest to the overall prevalence in the derivation cohort (13.8% vs. 12.6%), a scoring of ≤2 was categorized as “Average Risk” **(AR)**. Subjects with scores ≥3 had proportions higher than the overall prevalence, and hence were designated as “High Risk” **(HR)**. For synchronous PN/DN, a score of 2 was also chosen as the cut-off point to differentiate between AR and HR, since its proportion was closest to the overall prevalence in the derivation cohort (3.3% vs. 4.7%). The Cochran-Armitage test of trend showed that the prevalence of PN and PN/DN increased significantly with higher scores in both scoring systems (both p < 0.001).

### Model validity

For the risk prediction system for PN, 84.1% of the derivation cohort was classified as AR and 15.9% as HR (Table 8). In the derivation cohort, the prevalence of PN was 10.9% in the AR group and 21.0% in the HR group, similar to the respective figures in the validation cohort. For synchronous PN/DN, the corresponding prevalence was 2.2% (AR) and 8.5% (HR), also similar to those of the validation cohort.

**Figure 1:**
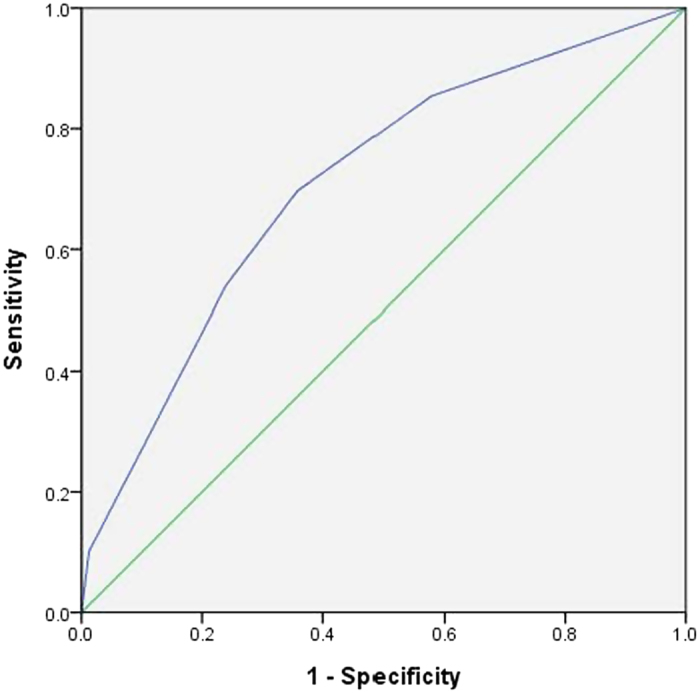
The Receiver Operating Characteristic (ROC) curve for prediction of proximal neoplasia. (The Area Under the Curve (AUC) was 0.71).

**Figure 2:**
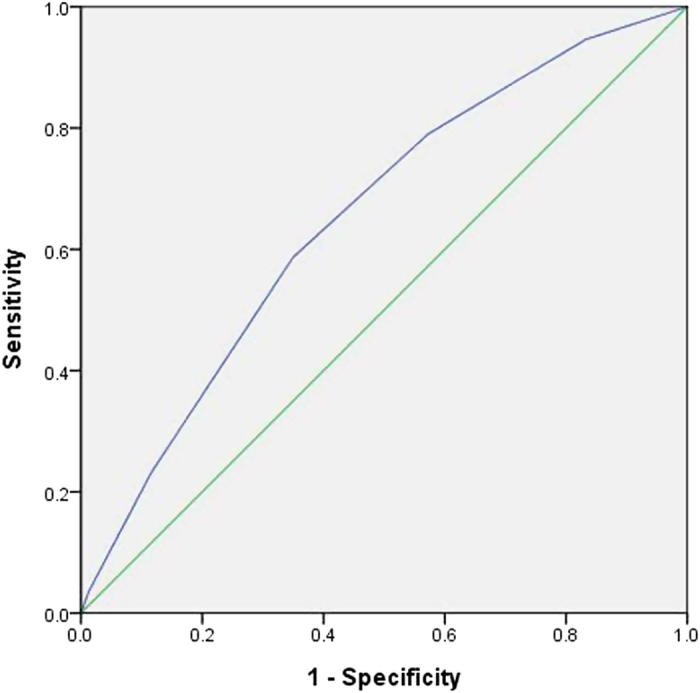
The Receiver Operating Characteristic (ROC) curve for prediction of synchronous proximal and distal neoplasia. (The Area Under the Curve (AUC) was 0.65).

For prediction of PN alone and synchronous PN/DN, the c-statistics for the risk score was 0.71 (0.02) and 0.65 (0.02), respectively. The ROC curves were shown in Figs 1 and 2. When compared with participants in the AR group, subjects in the HR group had a significantly higher risk of PN (AOR 1.51, 95% C.I. 1.24–1.84) and synchronous PN/DN (AOR 2.52, 95% C.I. 1.87–3.41). The Hosmer-Lemeshow goodness-of-fit statistic evaluating the reliability of both validation sets had p values of 0.381 and 0.382, respectively, indicating a close match between predicted risk and real risk. Application of the prediction algorithm would miss 9.1% of proximal neoplasia (178 out of 1,946 subjects) and 1.4% of synchronous proximal and distal neoplasia (27 out of 1,946 subjects).

## Discussion

### Statement of principal findings

This study has devised and validated two simple clinical risk scoring systems for predicting PN and synchronous PN/DN, respectively, in asymptomatic subjects. There is a trend toward higher detection rate of proximal neoplasia with increasing scores. The instrument is simple and easy to use, and the risk prediction only requires basic clinical information. The scoring system is particularly suited for patients who are keen to obtain more comprehensive information about their risks, where their screening choice could be facilitated. We recommend subjects who scored ≥3 points in either system may choose colonoscopy, whereas those with scores ≤2 could select FS as the primary CRC screening test. In should be noted that the prevalence of synchronous PN/DN among subjects who scored 5 was 28.1%, where colonoscopy is strongly indicated. The use of this scoring system in clinical practice is consistent with the advocates from the Institute of Medicine^28^ and the US Preventive Services Task Force^29^, where shared decision making should be promoted in screening practices. Besides, its application could rationalize the use of colonoscopy in circumstances where the risk of proximal lesions should be adequately high to warrant the procedure. These findings could also inform policy-makers at the macro level, especially when the characteristics of eligible residents in population-based screening programmes are known. The use of this tool could have a substantial public health implication, as resources to equip colonoscopy and FS capacity could be more accurately planned. Some variables, such as family history of CRC, were significantly associated with PN but not with synchronous PN/DN, and this might be explained by the relatively small sample size among those with synchronous PN/DN and the risk factors at the same time.

### Relationship with literature

From a thorough literature review, there are only few studies which devised a validated scoring system for prediction of PN. Imperiale and colleagues have developed a seven-point risk stratification tool based on age, gender, and distal findings on FS from a company-based programme of screening colonoscopy in Indianapolis^16^. A methodology similar to the present study was used. All three variables were found to be independent predictors and formed a derivation cohort, and the outcome of interest is proximal advanced neoplasia^30^. However, when the scoring system was later evaluated in an average-risk asymptomatic cohort in Boston, it was reported that the clinical index has limited ability to differentiate low from intermediate risk white, black and Hispanic patients for PN (c-statistics < 0.07)^17^. Another large-scale study including more than 10,000 adults concluded that proximal advanced neoplasia is a function of age and gender only^18^. Yet another evaluation was conducted in California involving more than 2,900 asymptomatic subjected aged ≥5 0 years undergoing colonoscopy as a follow up to screening sigmoidoscopy. It was found that age, family history and distal findings were significant predictors of proximal advanced neoplasia^19^. When compared with these existing tools, our scoring system is unique as it does not rely on distal findings for risk prediction, yet it has high accuracy and predictive ability. A possible difference in discriminative ability between the present scoring system and the existing ones^16^,^17^,^18^,^19^,^30^ may be due to differences in subject ethnicity and the outcomes of interest – as we studied colorectal neoplasia instead of advanced neoplasia in the proximal colon. The exact reasons for the difference remain to be further explored. In addition, it is noteworthy that whilst some previous studies demonstrated higher prevalence of proximal neoplasia in men than in women^16^,^18^,^30^, some studies showed that women had higher risk for proximal neoplasia^31^,^32^,^33^. The discrepancy in gender difference among these studies could be due to different study designs (e.g. biomathematical modelling^31^ vs. the use of cancer registries^32^ vs. prospective recruitment of screening participants^16^,^17^,^18^,^19^,^30^,^33^). There have also been speculations that sociocultural barriers within female subjects were present to delay screening and diagnosis; as well as different nutrient metabolism and dietary practices when compared with male subjects. Although these have been identified as factors which might influence the risk of proximal neoplasia in different populations^33^, the exact reasons for the gender-specific discrepancy will need to be further explored.

It was found that the adjusted odds ratio for proximal neoplasia among those with IHD was the highest (2.2) when compared with other risk factors (1.3–1.8). Many of the risk factors for IHD, namely smoking, alcohol drinking, hypertension, diabetes, and obesity which were entities within the metabolic syndrome were also risk factors for colorectal neoplasia. One biologically plausible explanation includes the fact that when IHD develops in screening participants, they might have been exposed to all these risk factors for a prolonged period of time which could potentially explain the higher odds among those with IHD. Future studies should evaluate the relative risks for proximal neoplasia conferred by established IHDs compared with healthy individuals.

### Strengths and Limitations

This is the first study which devised a scoring system to predict PN in a large cohort of asymptomatic individuals. It was conducted in an Asian Chinese population, which may be extrapolated to the 1.2 billion Chinese populations in the globe, due to subject homogeneity. A few limitations should however be addressed here. Firstly, we included self-referred screening participants in this study. Their health-seeking behavior and health consciousness might be different from the general public. Nevertheless, it is impractical to recruit participants by simple random sampling of the entire population, as the anticipated refusal rate will be high. Secondly, we invited screening participants aged between 50 to 70 years, and the utility of this system might not extend to subjects outside this age range. In addition, there are other potential risk factors which have not been included in the modeling, including physical activity level^34^, dietary intake of saturated fat, red meat and fibre^35^,^36^, as well as waist circumference which has recently shown to be an accurate predictor of colorectal neoplasia^37^. However, these variables are difficult to measure accurately, and could be subject to recall biases. Furthermore, we have used BMI as a measure of obesity and other anthropometric measurements including waist circumference, waist-to-hip ratio and body fat distribution could be additional parameters to enhance the predictive capability of the model. Finally, although IHD was found to be a novel predictive component of proximal neoplasia in this study which is compatible with a recent evaluation^38^, the present system used self-reported measures.

### Study implications and future research

In summary, we have devised and validated a clinical scoring system for prediction of PN in a Chinese population. It is anticipated that its use in clinical practice could assist physicians to risk stratify subjects for colorectal cancer screening, and offer a choice between FS and colonoscopy-based on individual risk of proximal neoplasia. Prospective screening participants could observe the possible risks of missing proximal neoplasia, and physicians could base on these figures to facilitate a more thoroughly informed, shared decision making discussion with their patients. Future research should evaluate the scoring system in other countries with different ethnicity and distribution of colorectal neoplasia. The projected cost-effectiveness, acceptability, and practicality to implement this prediction tool in screening practices should be further addressed.

## Additional Information

**How to cite this article**: Wong, M. C.S. *et al.* Validation of a risk prediction score for proximal neoplasia in colorectal cancer screening: a prospective colonoscopy study. *Sci. Rep.* **6**, 20396; doi: 10.1038/srep20396 (2016).

## Figures and Tables

**Table 1:** Characteristics of patients in the derivation and validation populations.

	Derivation cohort N = 2,000(random sample)	Validation cohortN = 3,789	**p-value**
Age (years), mean ± SD	57.80 ± 4.89	57.70 ± 4.92	0.438
BMI (kg/m^2^), mean ± SD	23.65 ± 3.18	23.47 ± 3.19	0.043
Gender, male, N(%)	943 (47.2)	1,823 (47.0)	0.911
Ever Smoking, N(%)	172 (8.7)	288 (7.5)	0.102
Alcohol consumption, N(%)	197 (9.8)	364 (9.4)	0.564
Diabetes mellitus, N(%)	141 (7.0)	293 (7.6)	0.484
Family history present for a first-degree relative, N(%)	292 (14.6)	525 (13.5)	0.263
Hypertension, N(%)	474 (23.7)	864 (22.3)	0.217
IHD, N(%)	35 (1.8)	63 (1.6)	0.721
COAD, N(%)	16 (0.8)	25 (0.6)	0.497
Stroke, N(%)	13 (0.6)	25 (0.6)	0.980
Cirrhosis, N(%)	4 (0.2)	4 (0.1)	0.340
GERD, N(%)	102 (5.1)	202 (5.2)	0.860
Use of Non-steroidal anti-inflammatory drugs, N(%)	83 (4.2)	186 (4.8)	0.262
Use of Aspirin, N(%)	44 (2.2)	95 (2.4)	0.551
Normal/Diverticulum/Colitis/Others	1,094 (54.7)	2,146 (55.3)	0.649
Hyperplastic polyps	170 (8.5)	314 (8.1)	0.592
Colorectal neoplasia* N(%)	645 (32.2)	1,264 (32.6)	0.795
Cancer, N(%)	8 (0.4)	17 (0.4)	0.831
Advanced neoplasia, N(%)	122 (6.1)	232 (6.0)	0.856
Proximal neoplasia N(%)	253 (12.6)	502 (12.9)	0.752
Distal neoplasia N(%)	298 (14.9)	587 (15.1)	0.813
Proximal and distal neoplasia N(%)	94 (4.7)	175 (4.5)	0.743

BMI: Body Mass Index; IHD: Ischemic Heart Disease; COAD: Chronic Obstructive Airway Disease; GERD: Gastro-Esophageal Reflux Disease.

^*^Colorectal neoplasia include adenoma, advanced adenoma and cancer. Advanced neoplasia is defined as colorectal cancer, or any colorectal adenoma which has a size of ≥10 mm in diameter, high grade dysplasia, villous or tubulovillous histologic characteristics, or any combination thereof.

**Table 2:** Prevalence of proximal neoplasia and synchronous proximal and distal neoplasia in the derivation cohort by risk factors.

	All subjectsPrevalence (%)	Proximal neoplasiaN = 2,000		Synchronous proximal anddistal neoplasia N = 2,000	
		**Prevalence (%)**	**p-value**	**Prevalence (%)**	**p-value**
Gender					
Male	943 (47.2)	145 (15.4)	**0.001**	68 (7.2)	**<0.001**
Female	1,057 (52.8)	108 (10.2)		26 (2.5)	
Age					
50–55	741 (37.0)	67 (9.0)	**0.001**	23 (3.1)	**<0.001**
56–60	689 (34.4)	95 (13.8)		29 (4.2)	
61–65	396 (19.8)	65 (16.4)		23 (5.8)	
66–70	174 (8.7)	26 (14.9)		19 (10.9)	
Family history present for a first-degree relative					
Present	292 (14.6)	48 (16.4)	**0.035**	12 (4.1)	0.606
Absent	1,708 (85.4)	205 (12.0)		82 (4.8)	
Smoking					
Never	1,801(91.3)	220 (12.2)	0.271	77 (4.3)	**0.008**
Current or past	172 (8.7)	26 (15.1)		15 (8.7)	
Alcohol drinking ≥2 times/week					
No	1,803 (90.2)	225 (12.5)	0.487	78 (4.3)	**0.017**
Yes	197 (9.8)	28 (14.2)		16 (8.1)	
Diabetes					
No	1,859 (93.0)	230 (12.4)	0.175	85 (4.6)	0.327
Yes	141 (7.0)	23 (16.3)		9 (6.4)	
BMI*					
<25	1,358 (69.8)	153 (11.3)	**0.013**	52 (3.8)	**0.017**
≥25 (Obesity)	588 (30.2)	90 (15.3)		37 (6.3)	
Hypertension					
No	1,526 (76.3)	185 (12.1)	0.203	64 (4.2)	0.055
Yes	474 (23.7)	68 (14.3)		30 (6.3)	
IHD					
No	1,965 (98.2)	244 (12.4)	**0.019**	93 (4.7)	0.603
Yes	35 (1.8)	9 (25.7)		1 (2.9)	
COAD					
No	1,984 (99.2)	253 (12.8)	0.126	93 (4.7)	0.769
Yes	16 (0.8)	0 (0)		1 (6.2)	
Stroke					
No	1,987 (99.4)	252 (12.7)	0.590	94 (4.7)	0.422
Yes	13 (0.6)	1 (7.7)		0 (0)	
Cirrhosis					
No	1,996 (99.8)	252 (12.6)	0.457	94 (4.7)	0.657
Yes	4 (0.2)	1 (25.0)		0 (0)	
GERD					
No	1,898 (94.9)	242 (12.8)	0.561	90 (4.7)	0.703
Yes	102 (5.1)	11 (10.8)		4 (3.9)	
Use of Non-steroidal anti-inflammatory drugs					
No	1,917 (95.8)	244 (12.7)	0.613	90 (4.7)	0.958
Yes	83 (4.2)	9 (10.8)		4 (4.8)	
Use of Aspirin					
No	1,956 (97.8)	246 (12.6)	0.511	93 (4.8)	0.442
Yes	44 (2.2)	7 (15.9)		1 (2.3)	

BMI: Body Mass Index; IHD: Ischemic Heart Disease; COAD: Chronic Obstructive Airway Disease; GERD: Gastro-Esophageal Reflux Disease. *Colorectal neoplasia include adenoma and advanced neoplasia. Advanced neoplasia is defined as colorectal cancer, or any colorectal adenoma which has a size of ≥10 mm in diameter, high grade dysplasia, villous or tubulovillous histologic characteristics, or any combination thereof.

^*^54 missing BMI.

**Table 3:**
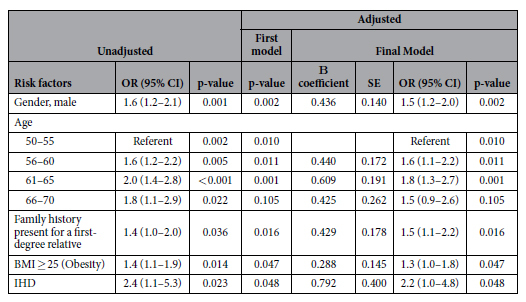
Univariate and multivariate predictors of proximal neoplasia in the derivation cohort.

BMI: Body Mass Index. *Colorectal neoplasia include adenoma and advanced neoplasia. Advanced neoplasia is defined as colorectal cancer, or any colorectal adenoma which has a size of ≥10 mm in diameter, high grade dysplasia, villous or tubulovillous histologic characteristics, or any combination thereof.

**Table 4:**
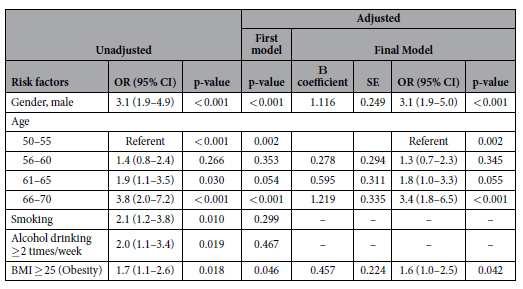
Univariate and multivariate predictors of synchronous proximal & distal neoplasia in the derivation cohort.

BMI: Body Mass Index. *Colorectal neoplasia include adenoma and advanced neoplasia. Advanced neoplasia is defined as colorectal cancer, or any colorectal adenoma which has a size of ≥10 mm in diameter, high grade dysplasia, villous or tubulovillous histologic characteristics, or any combination thereof.

**Table 5:** Colorectal Screening score for prediction of risk for proximal neoplasia.

**Risk factor**	**Criteria**	**Points**
Age	50–55	0
	56–70	1
Gender	Female	0
	Male	1
Family history present for a first-degree relative	Absent	0
	Present	1
BMI	<25	0
	≥25 (Obesity)	1
IHD	Absent	0
	Present	1

BMI: Body Mass Index.

*Colorectal neoplasia include adenoma and advanced neoplasia. Advanced neoplasia is defined as colorectal cancer, or any colorectal adenoma which has a size of ≥10 mm in diameter, high grade dysplasia, villous or tubulovillous histologic characteristics, or any combination thereof.

**Table 6:** Colorectal Screening score for prediction of risk for synchronous proximal and distal neoplasia.

**Risk factor**	**Criteria**	**Points**
Age	50–55	0
	56–65	1
	66–70	2
Gender	Female	0
	Male	2
BMI	<25	0
	≥25 (Obesity)	1

BMI: Body Mass Index.

*Colorectal neoplasia include adenoma and advanced neoplasia. Advanced neoplasia is defined as colorectal cancer, or any colorectal adenoma which has a size of ≥10 mm in diameter, high grade dysplasia, villous or tubulovillous histologic characteristics, or any combination thereof.

**Table 7:** Distribution of number of subjects for each score category in the derivation cohort.

**Score**	**No. of subjects (%)**	No. of subjects withproximal neoplasia (%)	No. ofsubjects (%)	No. of subjects with proximaland distal neoplasia (%)	
0	258 (13.3)	16 (6.2)	308 (15.8)	5 (1.6)	Average risk (AR)
1	670 (34.4)	64 (9.6)	489 (25.1)	8 (1.6)	
2	708 (36.4)	98 (13.8)	422 (21.7)	14 (3.3)	
3	272 (14.0)	57 (21.0)	460 (23.6)	39 (8.5)	High risk (HR)
4	38 (2.0)	8 (21.1)	235 (12.1)	14 (6.0)	
5	0 (0)	0 (0)	32 (1.6)	9 (28.1)	

^*^Colorectal neoplasia include adenoma and advanced neoplasia. Advanced neoplasia is defined as colorectal cancer, or any colorectal adenoma which has a size of ≥10 mm in diameter, high grade dysplasia, villous or tubulovillous histologic characteristics, or any combination thereof.

**Table 8:** Prevalence of proximal neoplasia and proximal advanced neoplasia by risk tier.

**Proximal neoplasia**						**Synchronous proximal and distal neoplasia**				
**Derivation cohort**			**Validation cohort**			**Derivation cohort**		**Validation cohort**		
Risk Tier(Risk Score)	No. ofSubjects (%)	ProximalNeoplasia* (%)(95% CI)	No. ofSubjects (%)	ProximalNeoplasia (%)(95% CI)	Relative Risk(95% C.I.)	No. ofSubjects (%)	Proximal Neoplasia* (%)(95% CI)	No. ofSubjects (%)	Proximal Neoplasia (%)(95% CI)	Relative Risk(95% C.I.)
Average Risk(0–2)	1,636 (84.1)	178 (10.9) (9.4–12.5)	3,231 (85.0)	388 (12.0) (10.9–13.2)	1.00	1,219 (62.6)	27 (2.2) (1.5–3.3)	2,431 (64.0)	69 (2.8) (2.2–3.6)	1.00
High Risk(3–5)	310 (15.9)	65 (21.0) (16.7–26.0)	569 (15.0)	103 (18.1) (15.1–21.6)	**1.51 (1.24**–**1.84) P < 0.001**	727 (37.4)	62 (8.5) (6.7–10.9)	1,369 (36.0)	98 (7.2) (5.9–8.7)	**2.52 (1.87**– **3.41) P < 0.001**
Total	1,946 (100)	243 (12.5) (11.1–14.1)	3,800 (100)	491 (12.9) (11.9–14.0)		1,946 (100)	89 (4.6) (3.7–5.6)	3,800 (100)	167 (4.4) (3.8–5.1)	

*Colorectal neoplasia include adenoma and advanced neoplasia. Advanced neoplasia is defined as colorectal cancer, or any colorectal adenoma which has a size of ≥10 mm in diameter, high grade dysplasia, villous or tubulovillous histologic characteristics, or any combination thereof.
